# Challenges in diagnosis and treatment of recurrent ameloblastic carcinoma in the maxilla: case report and narrative review of current literatures

**DOI:** 10.1093/jscr/rjaf153

**Published:** 2025-04-06

**Authors:** Nouf A Almatrafi, Mohammad Alessa, Sherif Abdelmonim, Mohammed Alqaddi, Mohammad G M Raslan, Mohamad M El-Labban, Besada S A Fanous

**Affiliations:** Pharmacy Department, Saudi German Hospital Makkah, Ibrahim Al-Khalil Road, Waliu Aleahd District, Makkah 21955, Saudi Arabia; Head and Neck & Skull Base Health Centre, King Abdullah Medical City, Al Mashair Street, Al Aziziyah Neighborhood, Makkah 57657, Saudi Arabia; Head and Neck & Skull Base Health Centre, King Abdullah Medical City, Al Mashair Street, Al Aziziyah Neighborhood, Makkah 57657, Saudi Arabia; Otolaryngology, Head, and Neck Surgery Department, Faculty of Medicine, Ain Shams University, 38 Abbassia Street, Cairo 1181, Egypt; Head and Neck & Skull Base Health Centre, King Abdullah Medical City, Al Mashair Street, Al Aziziyah Neighborhood, Makkah 57657, Saudi Arabia; General Authority of Health Care, Corniche El Nile, Maadi, Cairo, Egypt; Faculty of Medicine, Port Said University, 23 December Street, Al-Zohour, Port Said 42534, Egypt; Ali Bin Ali Hospital, Mohammed Rashid Rida Street, Al Aziziyah District, Riyadh 14515, Saudi Arabia

**Keywords:** ameloblastic carcinoma, maxilla, treatment, diagnosis, recurrence

## Abstract

Ameloblastic carcinoma (AC) is a malignant transformation of benign ameloblastoma originating from epithelial cells of tooth enamel and has the potential for metastasis. A 42-year-old male experienced three recurrences of AC and hospitalized for surgical treatment. He was initially diagnosed as maxillary nasal sinus squamous cell carcinoma. However, the patient experienced a third recurrence, and additional examinations revealed the diagnosis of AC. The patient underwent right subtotal maxillectomy through Weber Ferguson incision with Dieffenbach extension. Followed by a post-operative radiotherapy course. This case report discusses the histopathological findings and current challenges of diagnosing and managing the recurrent AC. The findings suggest that the use of chemotherapy, radiotherapy, or a combination to surgical interventions is still restricted. Evidence available is insufficient to draw definitive conclusions regarding their safety and efficacy of different treatment modalities in AC treatment.

## Introduction

Malignant ameloblastic neoplasms are extremely rare and account for 1.6%–2.2% of all odontogenic tumors [1]. Odontogenic tumors include metastasizing ameloblastomas and ameloblastic carcinoma (AC). AC is a malignant transformation of benign ameloblastoma originating from the epithelial cells of the tooth enamel and has metastatic potential [2–4]. AC was first introduced by Elzay in 1982 and described by Shafer *et al.* in 1983 as an ameloblastoma with malignant histological features. However, the World Health Organization (WHO) recognized the AC as a separate tumor in 2005 [2, 3]. The latest update of WHO classification of Head and Neck tumors (2022) subclassified the AC into primary and secondary types (intraosseous and peripherally differentiated) [5]. This case report discusses the histopathological results of recurrent AC in the maxilla.

## Case report

**Figure 1:**
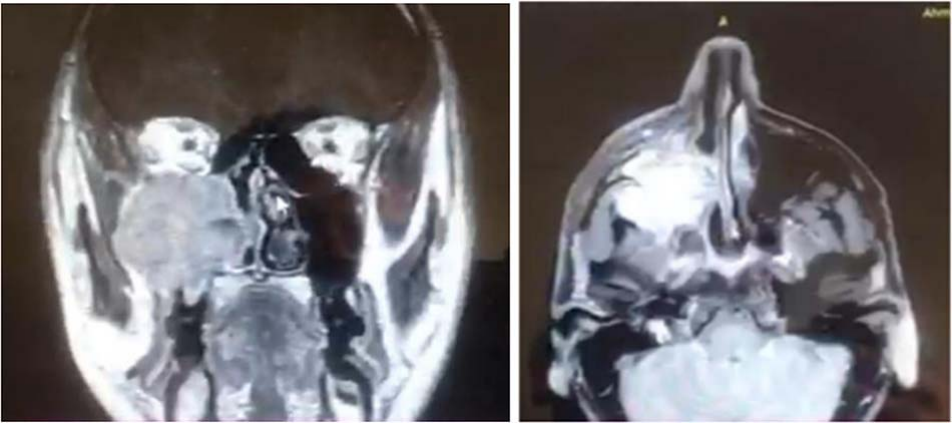
MRI image of right maxillary sinus-centered AC.

**Figure 2:**
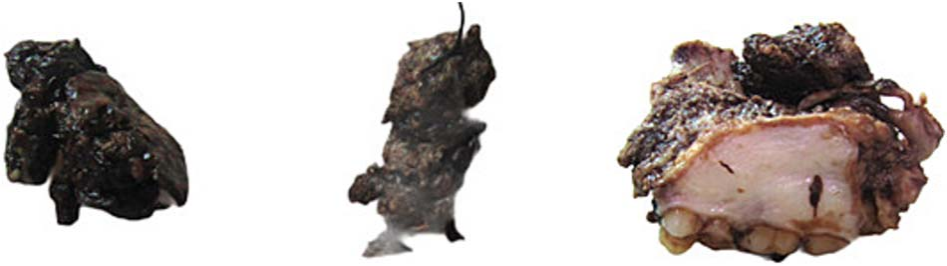
The excised ameloblastic tumor mass of the right maxillary sinus.

**Figure 3:**
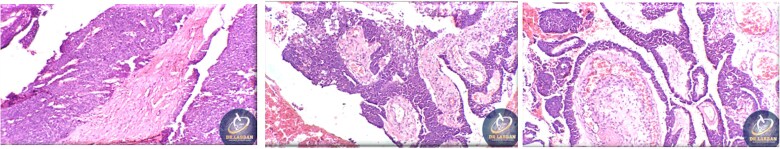
Histopathological findings of specimen at lower magnification.

**Figure 4:**
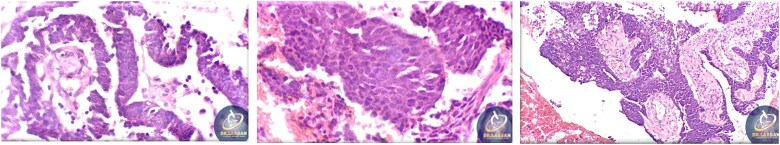
Histopathological findings of specimen at higher magnification.

**Figure 5:**
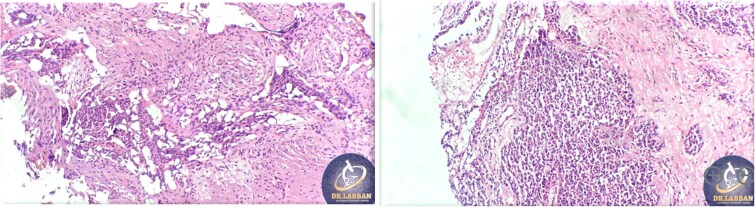
Tumor extension to the orbital floor and buccal mucosal surgical margin.

**Figure 6:**
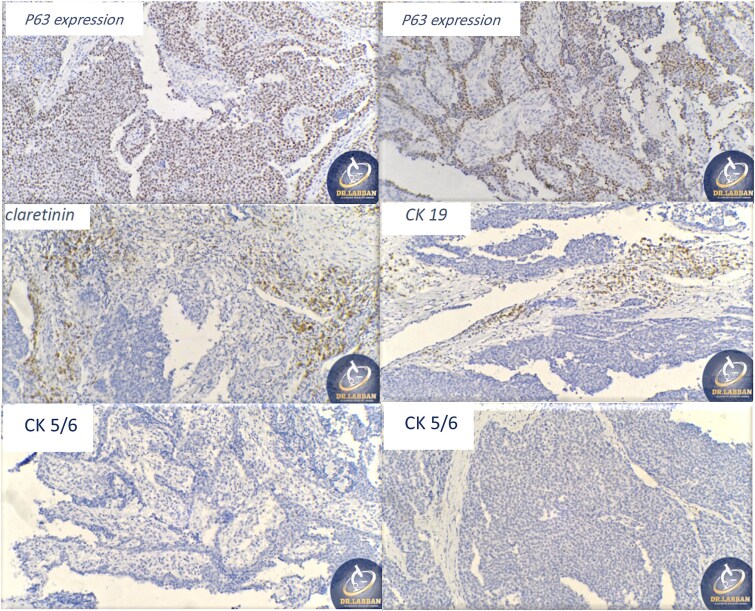
Ancillary studies for CK 5/6, P63, CK19, SOX-2, SOX-10, calretinin, and Ki67.

A 42-year-old male previously diagnosed with maxillary nasal sinus squamous cell carcinoma (SCC) was hospitalized for surgical treatment of recurrent infiltrating AC. The patient experienced three recurrences with the initial treatment approach for the cancer as SCC, a non-keratinizing variant. Complete excision and curettage of the lining mucosa were performed. In the second recurrence, the patient underwent additional curettage and subsequently revealed the absence of tumor residues from the mucosa and submucosa. However, the patient experienced a third recurrence with rapid growth and infiltration to the floor of the right maxillary sinus, filling the sinus space, infratemporal fossa, pterygopalatine fossa, cheek buccal area, and orbital floor. The imaging studies were preformed revealing a neoplastic lesion centered on the right maxillary sinus measuring 10 × 5 × 4.5 cm with a low T1 signal, heterogeneous iso T2/STIR signal, and intense heterogeneous post-contrast enhancement (Fig. 1). AC was recognized as stage II based on Yang’s *et al.* classification system [6]. Right Subtotal maxillectomy was performed through a Weber Ferguson incision with a Dieffenbach extension, and the infiltration areas were cleared. The excised tumor mass from the right maxillary sinus with labeled surgical margins (palate, orbital floor, buccal, and pterygoid surgical margins) was subjected to permanent paraffin evaluation (Fig. 2). The Right hemi-maxillectomy measuring 6 × 4.5 × 4 cm, revealed one canine, two molars, and two premolars found in the alveolar arch. An indefinite, grayish-white, infiltrating, fungating mass, approximately (⁓6 × 4 × 2.5 cm), was observed in the maxillary sinus. The cut surface of the mass was grayish-white and firm, with skeletal muscle attachment and noticeable infiltration into the underlying periosteum of the alveolar arch. Furthermore, labeled tumor tissue fragments measuring 4.7 × 2 × 2.5 cm and multiple smaller pieces collectively measuring 4 × 4 × 2 cm, showing similar characteristics, were stored in the same container. Microscopic examinations were performed at different magnifications. At a low magnification, the specimen revealed the presence of an infiltrative tumor with a biphasic pattern. The Islands and epithelial cell sheets are embedded in the fibrous stroma. The tumor islands exhibited a basaloid appearance with peripheral palisading, reverse polarity, and central stellate reticulum-like cells, reminiscent of ameloblastoma. However, these neoplastic epithelial cells exhibited moderate cytological atypia and increased mitotic activity, which suggests of malignancy (Fig. 3). Higher magnification shows tumor cells with enlarged, pleomorphic nuclei, prominent nucleoli, and eosinophilic cytoplasm. Frequent mitotic figures, including atypical forms, were also observed. Peripherally, tumor islands comprise cells with hyperchromatic elongated nuclei exhibiting focal palisading. The central cells were loosely cohesive and exhibited a stellate reticulum-like morphology. Additionally, focal areas of necrosis, hemorrhage, and keratinization were prominent in the tumor islands, and the surrounding stroma appeared desmoplastic and encompassed moderate chronic inflammatory infiltrates (Fig. 4). Additionally, diffuse infiltration of tumor into the adjacent tissues was observed, including bone and soft tissues, and is associated with perineural and lymphovascular invasion. Tumor extension in the orbital floor and buccal mucosal surgical margins was evident. In contrast, the palatal and pterygoid surgical margins are free of tumor involvement (Fig. 5). Overall, the morphological features were consistent with a diagnosis of infiltrating AC. Furthermore, immunohistochemistry assay was performed using a Ventana BenchMark GX Auto-stainer, employing the Ventana I View DAB detection system and Ventana monoclonal antibodies. The sections were stained for CK 5/6, P63, CK19, SOX-2, SOX-10, calretinin, and Ki67. The results revealed that the tumor basaloid epithelial component was strongly positive for P63 and showed weak heterogeneous positive staining for CK19, calretinin, and SOX-2 (Fig. 6). Conversely, it was negative for CK5/6 and SOX-10 staining. The Ki67 labeled index within the epithelial component was ⁓14%, whereas the mesenchymal component was 8%. The post-operative radiotherapy at 66 Gy was successfully administered. The patient is currently under observation, with careful follow-up.

## Discussion

A literature review was performed to scrutinize previous studies on AC of the maxilla, and 93 cases were reported identified using the PubMed, Web of Science, and Google Scholar databases. The data collected from the articles included sex, ethnicity, age at tumor diagnosis, anatomic sites, tumor manifestations, symptoms, treatment (surgical intervention with or without radiation and/or chemotherapy), metastasis, follow-up duration, number of recurrences, time to recurrence, and length of survival. Table 1 lists the demographics of the cases published between 1948 and 2024. These findings highlight the challenges of AC diagnosis and management in clinical practice. According to literature, insufficient evidence was available on post-operative radiotherapy and/or chemotherapy integration (Table 2). To extrapolate, eight patients underwent a combination of post-operative radiotherapy and chemotherapy. Among them, three patients experienced recurrence and one patient had a residual tumor [7–10]. Similar to the combination results, the patients who received radiotherapy alone [11–14]. In addition, two patients who received post-operative chemotherapy alone experienced recurrence, and one of the two patients experienced two recurrences [15, 16]. Notably, the overall recurrence incidence is high, affecting 30% of patients, compared with the 34% of patients without recurrence. Furthermore, 24 months was the calculated median recurrence time in the reported cases of AC arising from the maxilla. The survival rate of the reported cases was 26.5 (57.7–12). Owing to the rarity of AC, its molecular pathology, molecular biology, and use of targeted therapy are still under investigation. In addition, the *BRAF*-*V600E* mutation incidence is high in osteogenic tumors, and reports discussing the utility of BRAF-targeting medications are limited, and another mutation that contributes in ameloblastoma development is SMO L412F [17–19]. Further studies on different diagnostic and treatment modalities are required to draw definitive conclusions regarding AC.

**Table 1:** Overview of patient demographics.

**Population**	N = 93
**Age** (years), Median (IQR)	61 (70–42)
**Sex**	
Male, n (%)	58 (62.3%)
Female, n (%)	27 (29%)
**Country**	
Asian	26 (27.9%)
African	13 (13.9%)
Caucasian	24 (25.8%)
Hispanic/Latino	11 (11.8%)
Other	4 (4.3%)
NR	15 (16.1%)
**Manifestation of tumor**	
Primary, n (%)	50 (53.7%)
Secondary, n (%)	14 (15%)
NR, n (%)	21 (22.5%)
**Sign and Symptoms**	
Pain Reported, n (%)	28 (30.1%)
Painless Reported, n (%)	28 (30.1%)
Swilling, n (%)	63 (67.7%)
Ulcer, n (%)	7 (7.5%)
Bleeding, n (%)	2 (2.1%)
epistaxis, n (%)	2 (2.1%)
Paresthesia, n (%)	3 (3.2%)
Trismus, n (%)	1 (1%)
Other, n (%)	9 (9.6%)
NR, n (%)	22 (23.6%)

Abbreviation: IQR, interquartile range; NR, not recorded.

**Table 2:** Overview of clinical features and treatment modalities.

**Population**	N = 93
**Anatomic Site**	
Anterior, n (%)	9 (9.6%)
Posterior, n (%)	50 (53.7%)
Both, n (%)	12 (12.9%)
NR, n (%)	14 (15%)
**Primary Intervention**	
Surgical Resection	79 (84.9%)
Radio/Chemotherapy	4 (4.3)
Other	2 (2.1%)
NR	8 (8.6%)
**Post-operative Course**	
yes	26 (27.9%)
No	53 (56.9)
**Type of Course**	
Chemotherapy	2 (7.9%)
Radiotherapy	16 (61.5%)
Both	8 (30.7%)
NR	8 (30.7%)
**Metastasis**	
Yes, n (%)	11 (11.7%)
No, n (%)	60 (63.8%)
NR, n (%)	14 (14.8%)
**Site of metastasis**	
L.N, n (%)	3 (27.2%)
Pulmonal, n (%)	10 (90.9%)
Hepatic, n (%)	3 (27.2%)
Cardiac, n (%)	1 (9%)
Cerebral, n (%)	1 (9%)
Cervical, n (%)	3 (27.2%)
Gastrointestinal, n (%)	1 (9%)
Vertebral, n (%)	1 (9%)
**Recurrence**	
None, n (%)	34 (36.5%)
Single, n (%)	26 (27.9%)
Multiple, n (%)	2 (2.1%)
Residual tumor, n (%)	5 (5.3%)
NR, n (%)	18 (19.3)
**Follow-up**	
Lost of Follow-up	2 (2.1%)
Death	18 (19.3%)
NR	23 (24.7%)
**Time to recurrence** (Months), Median (IQR)	24 (37–12)
**Survival Time** (Months), Median (IQR)	26.5 (57.7–12)
